# Exosomes derived from human mesenchymal stem cells preserve mouse islet survival and insulin secretion function

**DOI:** 10.17179/excli2020-2451

**Published:** 2020-08-03

**Authors:** Somayeh Keshtkar, Maryam Kaviani, Fatemeh Sabet Sarvestani, Mohammad Hossein Ghahremani, Mahdokht Hossein Aghdaei, Ismail H. Al-Abdullah, Negar Azarpira

**Affiliations:** 1 Department of Molecular Medicine, School of Advanced Technologies in Medicine, Tehran University of Medical Sciences, Tehran, Iran; 2 Transplant Research Center, Shiraz University of Medical Sciences, Shiraz, Iran; 3 Department of Translational Research and Cellular Therapeutics, Diabetes and Metabolism Research Institute, Beckman Research Institute of City of Hope, Duarte, CA/USA

**Keywords:** mesenchymal stem cell, exosome, islet, viability, function, VEGF

## Abstract

Islet cell death and loss of function after isolation and before transplantation is considered a key barrier to successful islet transplantation outcomes. Mesenchymal stem cells (MSCs) have been used to protect isolated islets owing to their paracrine potential partially through the secretion of vascular endothelial growth factor (VEGF). The paracrine functions of MSCs are also mediated, at least in part, by the release of extracellular vesicles including exosomes. In the present study, we examined (i) the effect of exosomes from human MSCs on the survival and function of isolated mouse islets and (ii) whether exosomes contain VEGF and the potential impact of exosomal VEGF on the survival of mouse islets. Isolated mouse islets were cultured for three days with MSC-derived exosomes (MSC-Exo), MSCs, or MSC-conditioned media without exosomes (MSC-CM-without-Exo). We investigated the effects of the exosomes, MSCs, and conditioned media on islet viability, apoptosis and function. Besides the expression of apoptotic and pro-survival genes, the production of human and mouse VEGF proteins was evaluated. The MSCs and MSC-Exo, but not the MSC-CM-without-Exo, significantly decreased the percentage of apoptotic cells and increased islet viability following the downregulation of pro-apoptotic genes and the upregulation of pro-survival factors, as well as the promotion of insulin secretion. Human VEGF was observed in the isolated exosomes, and the gene expression and protein production of mouse VEGF significantly increased in islets cultured with MSC-Exo. MSC-derived exosomes are as efficient as parent MSCs for mitigating cell death and improving islet survival and function. This cytoprotective effect was probably mediated by VEGF transfer, suggesting a pivotal strategy for ameliorating islet transplantation outcomes.

## Introduction

Type 1 diabetes comprises 5-10 % of all diabetic cases and is the consequence of the autoimmune destruction of pancreatic beta cells, giving rise to absolute insulin deficiency (Keshtkar et al., 2019[19]; You and Henneberg, 2016[43]). Islet transplantation is considered a promising treatment for type 1 diabetes patients who face severe periods of hypoglycemia (Saravanan et al., 2017[33]). Although the transplantation of islets is backed by encouraging results, some limitations exist, such as the shortage of organ donors and the loss of islets both in the pre-transplant culture period and subsequent to transplantation (Froud et al., 2005[10]). The disconnection of islets from the microvascular network and native niche leads to cell death, which continues during the culture period (Keshtkar et al., 2020[18]). Ahead of transplantation, the islet culture must adequately preserve the islet quality while the graft recipient is prepared and the isolated islets are transported to remote clinical centers for transplantation (Shapiro et al., 2017[37]). However, the culture period can lead to significant islet death triggered by hypoxia and oxidative stress (Keshtkar et al., 2020[18], 2019[19]). These insults induce apoptosis and decrease β-cell function, thereby exerting a negative impact on the outcome, sometimes even impeding transplantation (Arzouni et al., 2017[2]). 

The mitochondrial apoptosis pathway is recognized as a major cause of islet death. The members of the B-cell lymphoma-2 (BCL-2) family regulate the apoptosis pathway through interactions between pro-apoptotic and anti-apoptotic factors, including the BCL-2-associated agonist of cell death (BAD), BCL-2-associated X (BAX), and BCL-2 genes (Kaviani et al., 2019[14][16][15]). The balance between BAX and BCL-2 (i.e., the BAX/BCL-2 ratio) represents an index that determines the cellular fate in terms of survival or apoptosis (Greijer and Van der Wall, 2004[12]; Velmurugan et al., 2012[41]). Besides, apoptosis is regulated via pro-survival phosphoinositide 3-kinase (PI3K) signaling, which is known as a crucial regulator for the survival of isolated islets (Aikin et al., 2006[1]). The activation of PI3K signaling leads to the direct control of apoptosis via suppression of pro-apoptotic factors such as BAD (Downward, 2004[9]). 

The vascular endothelial growth factor (VEGF) is also known as a pro-survival and anti-apoptotic factor that preserves islet quality. This factor is expressed in pancreatic islets, but its expression is attenuated in isolated islets, which is associated with islet dysfunction and cell death (Cross et al., 2007[6]; Langlois et al., 2016[22]; Sigrist et al., 2003[38]). Moreover, VEGF contributes to the revascularization of transplanted islets, which comprises a vital step for the long-term survival of grafts (Miao et al., 2006[23]). 

Researchers have employed various methods of treatment with cytoprotective substances to protect isolated islets from apoptosis and improve islet survival and function, with one instance being the co-culturing of islets with supportive cells such as mesenchymal stem cells (MSCs) (Arzouni et al., 2017[2]; de Souza et al., 2017[8]; Scuteri et al., 2014[35]). Several studies have confirmed the positive effect of MSCs on islet viability and function through paracrine interactions and/or direct contact both in the culture period and after transplantation (Rackham et al., 2014[29]; Scuteri et al., 2014[35]; Yamada et al., 2014[42]). Some report that the protective effect of MSCs on cultured islets occurs via direct contact and the production of extracellular matrix molecules and annexin A1 (Arzouni et al., 2017[2]; Gamble et al., 2018[11]; Montanari et al., 2017[24]). This is while others suggest that indirect co-culture systems involving Transwell® inserts or conditioned media of MSCs (MSC-CM) could also inhibit apoptosis and promote islet viability and function through the secretion of trophic factors (de Souza et al., 2017[8]; Schive et al., 2017[34]; Yamada et al., 2014[42]). Recent evidence reveals that the paracrine functions of MSCs are not only mediated by the secretion of soluble factors but also by the release of extracellular vesicles including exosomes (Bian et al., 2019[3]; Keshtkar et al., 2018[17]). 

Exosomes are vesicles of endosomal origin spanning 40 to 150 nm in diameter, responsible for transporting functional RNA and proteins (Bian et al., 2019[3]; Valadi et al., 2007[40]). These small vesicles act as intercellular messengers and transfer their cargo to neighbor and recipient cells through endocytosis uptake, direct fusion, or receptor interaction, highlighting the potential role of exosomes in cell-to-cell communication (Keshtkar et al., 2018[17]). Exosomes are able to modify the transcriptome and proteome of recipient cells, thereby modulating apoptosis pathways and regulating differentiation and proliferation (Kore et al., 2019). Similar to the parent MSC, MSC-derived exosomes (MSC-Exo) offer cell protection against apoptosis. In fact, MSC-Exo are capable of repairing cells and protecting them from death according to *in vitro *and* in vivo* studies on cardiovascular diseases, acute kidney injury, brain damage, and lung injury (Keshtkar et al., 2018[17]; Konala et al., 2016[20]). However, the effect of MSC-Exo on isolated islets is yet to be investigated. 

The present study aimed to assess the impact of MSC-Exo on islet survival and function. In addition, we compared the effects of MSC, MSC-Exo, and MSC-conditioned media without exosomes (MSC-CM-without-Exo) on islets during the culture period. Subsequently, the possible underlying mechanisms behind the observed effects were investigated. 

## Method and Materials

### Mesenchymal stem cell (MSC) isolation and characterization

Umbilical cords were collected after informed consent was collected from mothers ahead of cesarean section delivery. Wharton's jelly-derived MSCs (WJ-MSCs) were isolated from the umbilical cords as previously described in the literature (Pirjali et al., 2013[27]; Shaer et al., 2014[36]). In brief, the small pieces of Wharton's jelly were placed in DMEM-F12 media (Gibco, Germany) along with 10 % FBS (Gibco, UK) and 1 % antibiotic (Sigma-Germany), ahead of incubation with 5 % CO_2 _at 37 °C. Cellular passage was done after cells reached 70 % confluence by 0.25 % trypsin- EDTA (Gibco, UK). For characterization, exosome isolation, and co-culture experimentation, WJ-MSCs from passage 3 were used. The isolated cells were assessed for surface markers CD105, CD44 and CD90, and CD34 (BioLegend, USA) with a FACSCalibur™ flow cytometer (Becton Dickinson, USA). Differentiation induction to adipocyte and osteocyte cells were performed with differentiation kits (Gibco, Germany), before the cells were stained with Oil red O (Sigma, Germany) to examine lipid vacuoles and Alizarin red S (Sigma, Germany) to identify calcium disposition. 

### Isolation and characterization of mesenchymal stem cell exosomes (MSC-Exo) 

Passage 3 of WJ-MSCs was cultured in RPMI-1640 medium supplemented with 10 % FBS and 1 % antibiotic to reach 80 % confluence. The cells were washed by PBS and cultured in RPMI-1640 containing 10 % exosome-depleted FBS (System Bioscience, USA) for the next three days. Then, exosome isolation was performed as described in the literature (Riazifar et al., 2019[31]). In brief, the conditioned media of MSCs (MSC-CM) were collected and centrifuged at 300 g for 10 min to eliminate any cells and debris. To remove the microvesicles, the supernatants were centrifuged by ultracentrifuge (XL-100K Ultracentrifuge, Beckman Coulter) at 16500 g for 20 min. The remaining supernatants were ultra-centrifuged at 120000 g for 90 min, and the exosome pellet was obtained. Conditioned media of MSCs without exosomes (MSC CM-without-Exo) were collected and stored at −80 °C ahead of further experimentation. The exosome pellet was subsequently re-suspended in PBS and re-centrifuged for further purification. Finally, the supernatant was discarded and the exosome pellet (MSC-Exo) was re-suspended in PBS and stored at -80 °C. All centrifuging and supernatant preparation processes were completed at 4 °C. 

The exosome content was measured with a BCA protein assay kit (Thermo Scientific Pierce, USA). A transmission electron microscope (TEM) was used (Zeiss, LEO 906E, Germany) for the observation of exosome morphology. Furthermore, the exosomes were characterized for specific surface markers including CD9, CD63, and CD81 (BD Bioscience, Belgium) by flow cytometry after they were incubated with magnetic beads to amplify the channel signal (Pospichalova et al., 2015[28]). 

### Mouse islet isolation and culture

Pancreases were obtained from Balb/C mice aged 8-12 weeks. All experiments were conducted in accordance with the Research Ethics and Animal Use Committee of the Shiraz University of Medical Sciences. Islet isolation was performed as previously described with some modifications (Sagheb et al., 2013[32]; Zongyi et al., 2017[46]). In brief, the pancreases were digested by collagenase P (Sigma, Germany) and purified by density gradient centrifugation. The collected islets were washed with Hank's solution and stained with dithizone (DTZ) (Sigma, Germany) for purity assessment and islet count. The number of islets was expressed in islet equivalents (IEQ). The isolated islets were cultured in RPMI-1640 (Sigma, Germany) supplemented with 10 % FBS, 1 % L-glutamine, and 1 % antibiotic/antimycotic in 5 % CO_2_ at 37 °C.

### Co-culture of mouse islets with mesenchymal stem cell exosomes (MSC-Exo) and mesenchymal stem cells (MSCs)

For the co-culture experiment, we used 400 IEQ of islets per well across four different groups. The first group included islets in isolation and was considered the control group. The second group was the MSC co-culture group; 1.5×10^5^ MSCs were cultured overnight before the conditioned medium was changed with fresh medium and the islets were added. For the third group (MSC-Exo group), 40 µg/ml of exosomes (Nie et al., 2018[25]) were added to the islets. These three groups were cultured in RPMI-1640 with 10 % exosome-depleted FBS and 1 % antibiotic. For the fourth group, the islets were cultured in the MSC-CM-without-Exo. All samples were incubated under 5 % CO_2_ at 37 °C for three days. Experimentation occurred in triplicates.

### Viability evaluation of cultured mouse islets

Staining with fluorescein diacetate (FDA) and propidium iodide (PI) (Sigma, Germany) was used for the evaluation of live and dead cells, respectively. Imaging was performed using a fluorescence microscope (CKX53, Olympus, Japan). The viability rate was expressed by the percentage of green area to the total area within intact islet (Keshtkar et al., 2019[19]).

### Apoptosis evaluation of cultured mouse islets

The terminal deoxynucleotidyl transferase-mediated dUTP nick end labeling (TUNEL) assay was performed for the assessment of apoptosis cells on islet slides using the Click-iT® Plus TUNEL assay kit (Life Technology, France) based on the manufacturer's protocol. Nuclear visualization was facilitated by a fluorescence microscope following DAPI (Sigma, Germany) counterstaining. The percentage of apoptotic islets was expressed by the percentage of TUNEL-positive cells out of all nuclei within each islet. 

### Gene expression evaluation by real-time PCR

To evaluate gene expression, RNA was extracted using an RNA-Sol isolation kit (Alphabio-Canada). For the MSC co-culture groups, the islets were separated from the MSCs under the microscope ahead of RNA extraction. Following the confirmation of RNA integrity, cDNA was synthesized with the PrimeScript TM RT Reagent Kit (Takara-Japan). We designed the following primers using the NCBI Primer-BLAST tool: mouse BCL-2 (F: 5´GGATAACGGAGGCTGGGATGC-3´; R: 5´-ATTTGTTTGGGGCAGGTTTGTCG-3´); mouse BAX (F: 5´-TTTTGCTACAGGGTTTCATCCAGG-3´; R: 5´-ATCATCCTCTGCAGCTCCATATTG-3´); mouse BAD (F: 5´GAGCAACATTCATCAGCAGGGACG-3´; R: 5´-GGTACGAACTGTGGCGACTCCG-3´); mouse PI3K (F: 5´-GCTGAGTGGCAAACGAGA- 3´; R: 5´-TGCGGTAGATGACACAGT-3´); mouse insulin (F: 5´-GCTCTCTACCTGGTGTGTGG-3´; R: 5´-GTGCCAAGGTCTGAAGGTC-3´); mouse VEGF (F: 5´-GTCCTCTCCTTACCCCACCT-3´; R: 5´-CACACACAGCCAAGTCTCCT-3´); mouse glyceraldehyde 3-phosphate dehydrogenase (GAPDH) (F: 5´-ACTGAGCAAGAGAGGCCCTA-3´; R: 5´-TATGGGGGTCTGGGATGGAA-3´); human VEGF (F: 5´-CTTCAAGCCATCCTGTGTGC-3´; R: 5´-ATCCGCATAATCTGCATGGTG-3´); and human GAPDH (F: 5´-GCTCATTTCCTGGTATGACAACG-3´; R: 5´-CTCTCTTCCTCTTGTGCTCTTG-3´) as a housekeeping gene. Real-time RT-PCR was used for the evaluation of relative gene expression with the SYBR® Premix Ex TaqTM II kit (Takara, Japan) and the Applied Biosystems StepOnePlus™ System (ABI, USA). The fold changes were calculated by 2^−^^ΔΔCT^ for each gene.

### Insulin secretion measurement of cultured mouse islets 

At the end of the culture period, the conditioned media of the islets were switched to RPMI 1640 without glucose (Gibco, Germany) containing 0.5 % BSA (Sigma, Germany) and 2.8 or 20 mM glucose (Sigma, Germany) for 1 hour. The culture media were collected, and insulin secretion was assessed via the ELISA assay (ELISA kit, BT LAB, China). The glucose stimulation index was calculated by dividing the amount of insulin secretion in the 20 mM glucose medium by the amount of insulin secretion in the parallel 2.8 mM glucose medium (Keshtkar et al., 2019[19]). 

### Measurement of vascular endothelial growth factor (VEGF) protein

Following exosome isolation, the amount of VEGF in the MSC-Exo and MSC-CM-without-Exo was measured using human VEGF ELISA kits (Life Technology, France). For exosome pellets, the RIPA buffer (Sigma, Germany) was used for protein extraction.

After the completion of the three-day culture period, human and mouse VEGF proteins were measured in the supernatant of cultured islets with specific human VEGF (Life Technology, France) and mouse VEGF ELISA kits (Sigma, Germany) based on the manufacturers' instructions. The results were represented as pg/ml. 

### Statistical analysis

All experiments were performed a minimum of three times. The results were expressed as mean ± SD. The comparisons between two groups were made by the unpaired student's t-test. To compare multiple groups, one-way analysis of variance (ANOVA) was used. Comparisons between the control group and the co-cultured groups were done with the Sidak test. The graphs were drawn in Graph Pad Prism software (Version 6, San Diego, California). A p-value below 0.05 was considered statistically significant.

## Results

### Isolation and identification of mouse islet with dithizone (DTZ)

**Figure 1:**
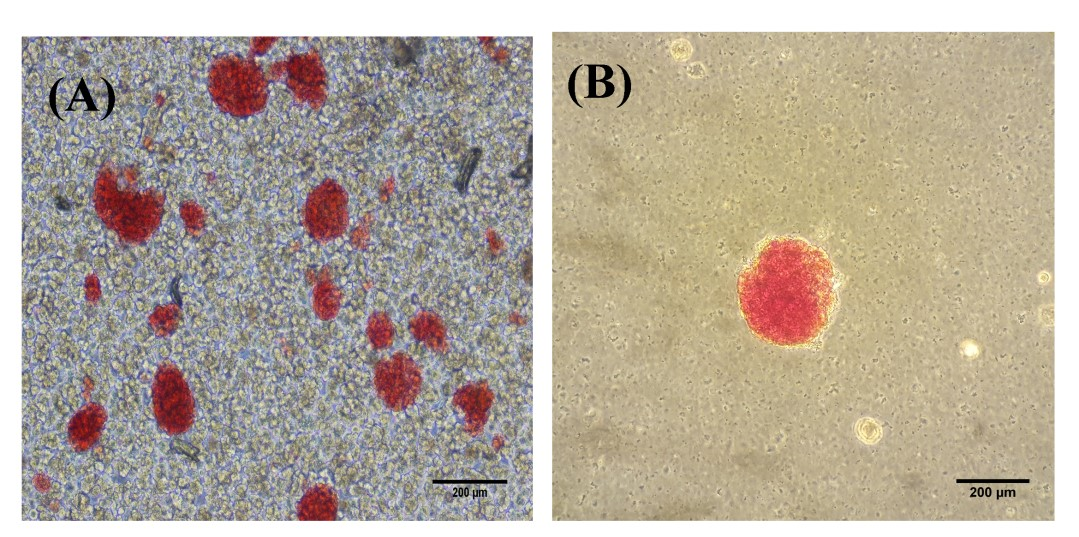
DTZ staining of the mouse islets after digestion (A) and purification (B). Scale bar = 200 µm

The isolated islets were identified with positive DTZ staining after enzymatic and mechanical digestion (Figure 1(Fig. 1)). The islets were stained as red-purple clamp cells and observed as spherical cell aggregates. During the subsequent three-day culture period, the islets maintained their shapes, especially in the treatment groups.

### Characterization and differentiation of Wharton's jelly-derived MSCs (WJ-MSCs)

**Figure 2:**
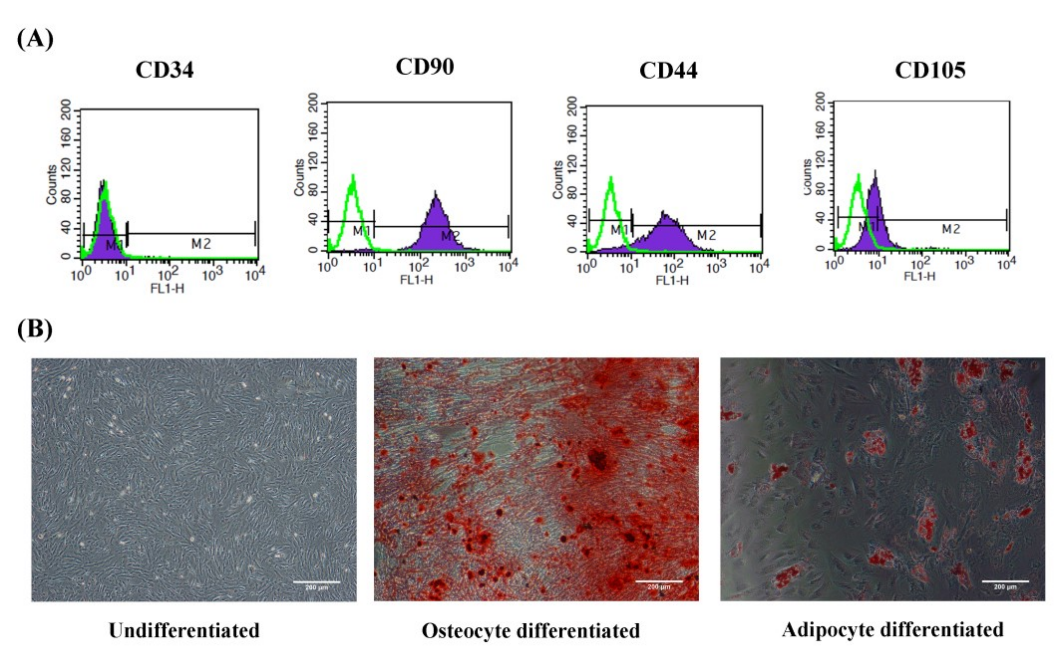
Characterization and differentiation of WJ-MSC. (A) Flow cytometry histogram surface of WJ-MSCs for CD34, CD44, CD105, and CD90 markers. Green histograms indicate isotype control, whereas purple histograms indicate the signals for each specific marker. (B) Differentiation of WJ-MSCs into osteocytes and adipocytes with Alizarin red S and Oil red O stains, respectively. WJ-MSCs: Wharton's Jell-derived mesenchymal stem cells; scale bar: 200 µm

The WJ-MSCs were strongly positive for surface markers CD44 and CD90, moderately positive for CD105, and negative for CD34 (Figure 2A(Fig. 2)). To evaluate the multipotency of the WJ-MSCs, adipocyte and osteocyte differentiation assays were performed. The presence of calcium deposits indicates the potential of the cells to differentiate into osteoblasts, while intracellular lipid vacuoles were observed as a prominent feature of adipocytes (Figure 2B(Fig. 2)).

### Characterization of mesenchymal stem cell exosomes (MSC-Exo)

**Figure 3:**
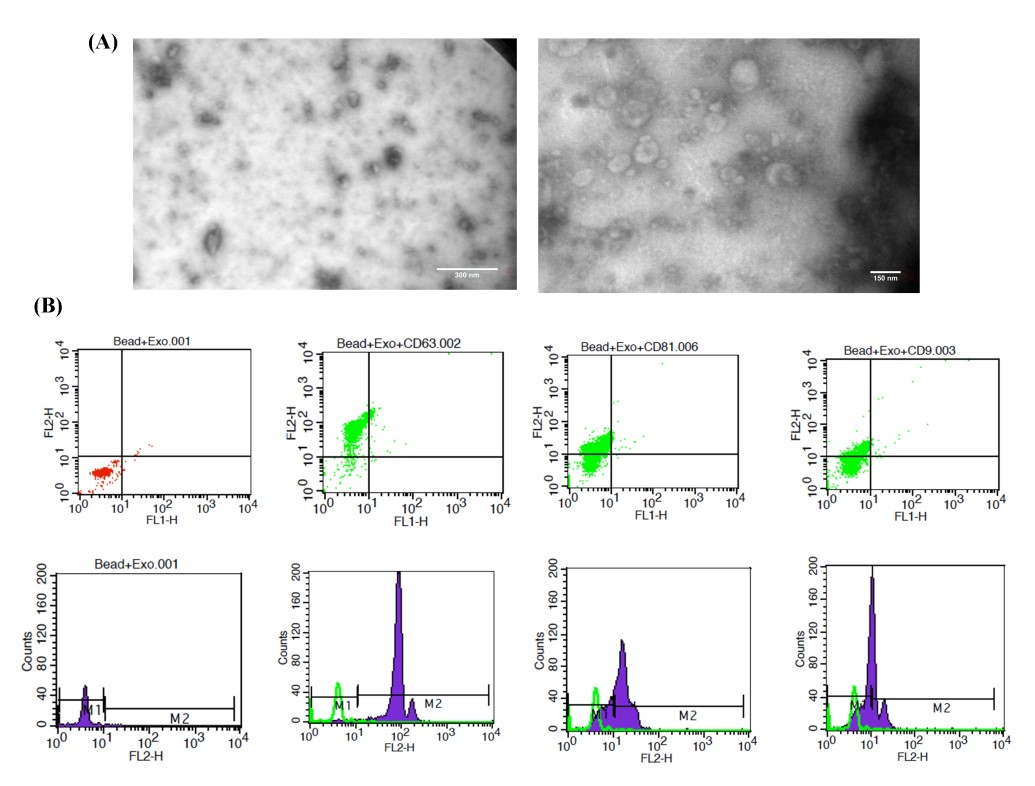
Characterization of MSC-Exo. (A) Morphology demonstrated by TEM. (B) Flow cytometry histogram surface of exosomes for the CD63, CD81, and CD9 markers. Green histograms indicate exosomes and beads without specific markers, whereas purple histograms indicate the signals for each specific marker. Scale bar: 300 nm and 150 nm. MSC-Exo: mesenchymal stem cell-derived exosomes

Exosomes were isolated from the conditioned media of WJ-MSCs by ultracentrifugation. Microscopy (TEM) revealed that the exosomes were spherically-shaped vesicles within the typical size range of 40-150 nm (Figure 3A(Fig. 3)). The expression of the conservative surface markers CD63, CD81, and CD9 on the isolated particles was confirmed by flow cytometry (Figure 3B(Fig. 3)).

### Mouse islet viability during the culture period

**Figure 4:**
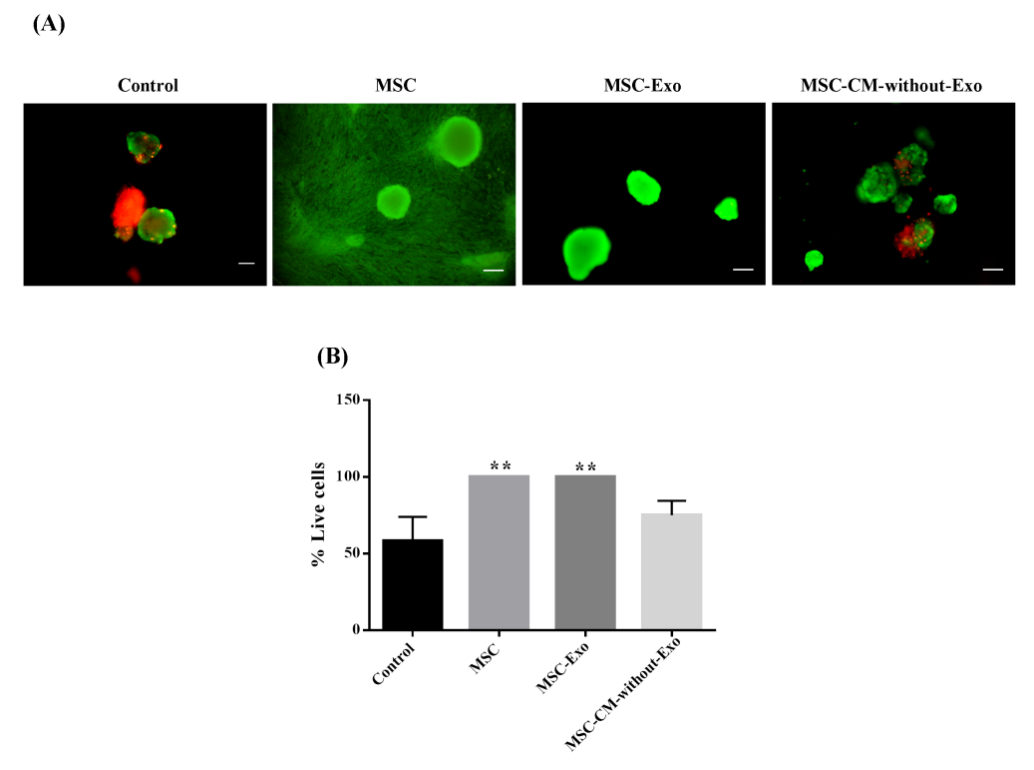
Mouse islet viability in the presence of MSCs, MSC-Exo, and MSC-CM-without-Exo. (A) Islets survival staining was done by fluorescein diacetate (green) for living cells and propidium iodide (red) for dead cells. (B) Viability of mouse islets in the different cultures. Scale bar: 200 μm, **P<0.01 MSC: mesenchymal stem cell; MSC-Exo: MSC-derived exosomes; MSC-CM-without-Exo: MSC-conditioned medium without exosomes

As shown in Figure 4(Fig. 4), mouse islet viability was assessed by FDA/PI staining. The percentage of viable islets in the MSC-Exo, MSCs, and MSC-CM-without-Exo groups was higher after the three-day culture period compared with the control group. Notably, the percentage of viable cells was significantly greater in the MSCs and MSC-Exo groups relative to the MSC-CM-without-Exo treatment. There was no statistically significant difference between the MSCs and MSC-Exo groups in this regard.

### Mouse islet apoptosis during the culture period 

**Figure 5:**
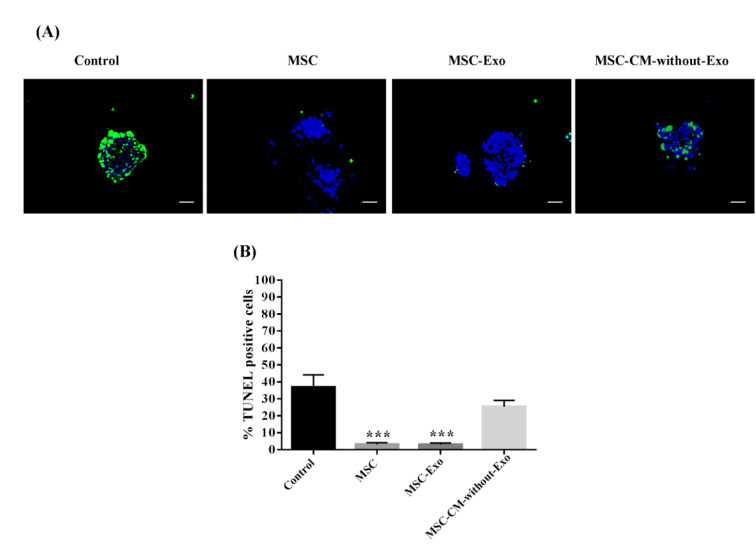
Mouse islet apoptosis in the presence of MSCs, MSC-Exo, and MSC-CM-without-Exo. (A) Apoptotic islets had green fluorescence and the nuclei were stained blue by DAPI dye. (B) The charts reveal the percentage of TUNEL-positive islets. Scale bar: 100 μm. *P<0.05 and ***P<0.001. MSC: mesenchymal stem cell; MSC-Exo: MSC-derived exosomes; MSC-CM-without-Exo: MSC-conditioned medium without exosomes

For the assessment of islet apoptosis, the TUNEL assay was employed. The percentage of TUNEL-positive cells in the control group was about 40 %, whereas the percentage of apoptotic cells decreased in the MSC-Exo, MSCs, and MSC-CM-without-Exo groups. The number of apoptotic cells was significantly fewer in the MSC-Exo and MSC groups relative to MSC-CM-without-Exo. There was no difference between the MSC-Exo and MSC treatments in this regard. The results showed that the MSC-Exo restored the viability of islets and decreased cell death in stress-exposed islets during the culture period, whereas the conditioned medium of MSCs without exosomes was not enough to rescue the islets from death (Figure 5(Fig. 5)).

### Changes in apoptotic-related gene expression of islets

**Figure 6:**
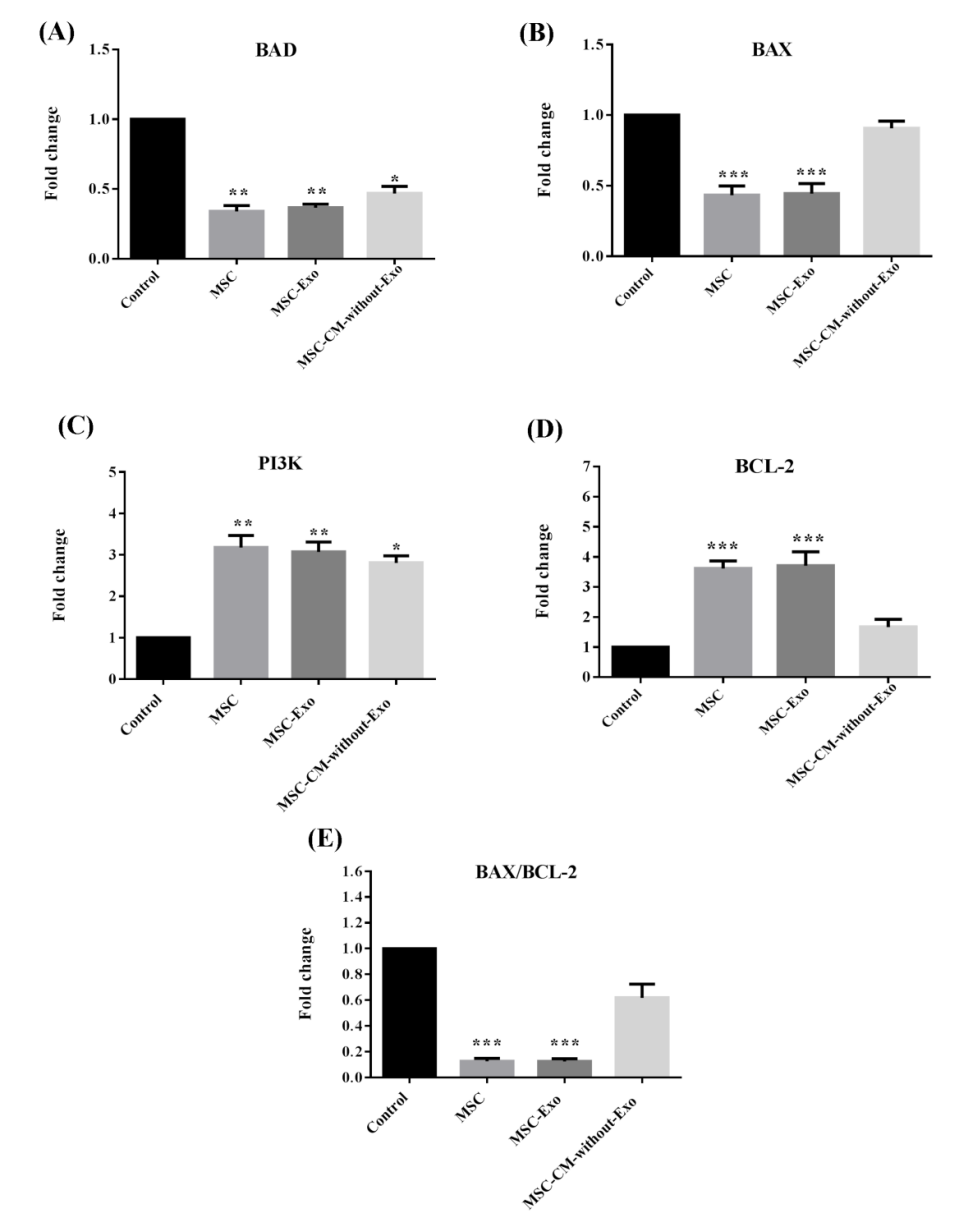
Gene expression of BCL-2, BAX, BAD, and PI3K as well as the BAX/BCL-2 ratio in mouse islets co-cultured with MSCs, MSC-Exo, and MSC-CM-without-Exo. *p<0.05, **p<0.01, and ***p<0.001. MSC: mesenchymal stem cell, MSC-Exo: MSC-derived exosomes; MSC-CM-without-Exo; MSC-conditioned medium without exosomes

The expression of pro-apoptotic genes including BAD and BAX along with the BCL-2 anti-apoptotic gene and the PI3K pro-survival gene was compared across the samples. The BAX/BCL-2 ratio was also calculated. In islets cultured with MSC-Exo or MSCs, the transcript expression levels of BAD and BAX were significantly downregulated, while the BCL-2 and PI3K genes were remarkably upregulated (Figure 6A-D(Fig. 6)). 

In the MSC-CM-without-Exo group, the reduction of BAD and enhancement of PI3K was significant, though the expression of BAX and BCL-2 did not alter significantly relative to the control group. Notably, the BAX/BCL-2 ratio fell significantly in the MSC-Exo and MSC treatments, but not in the MSC-CM-without-Exo group (Figure 6E(Fig. 6)). Therefore, although the MSC-CM-without-Exo exerted protective effects on islets during the culture period, this impact was not remarkable. 

### VEGF concentrations in the MSC-Exo and MSC-CM-without-Exo groups

**Figure 7:**
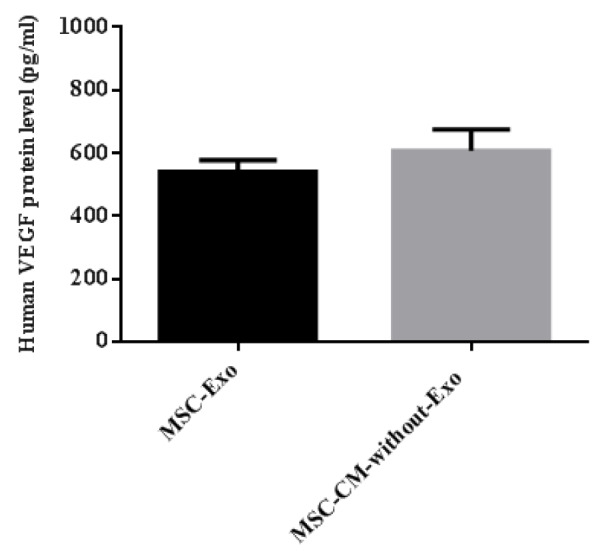
Evaluation of VEGF concentration in MSC-Exo compared with MSC-CM-without-Exo after ultracentrifugation. MSC: mesenchymal stem cell; MSC-Exo: MSC-derived exosomes; MSC-CM-without-Exo: MSC-conditioned medium without exosomes

The concentration of human VEGF was 539.3 ± 15.40 pg/mL in the MSC-Exo and 606.0 ± 28.16 pg/mL in the MSC-CM-without-Exo, suggesting that MSC-Exo contain a roughly equivalent amount of VEGF compared to MSC-CM-without-Exo (Figure 7(Fig. 7)).

### VEGF expression in mouse islets

**Figure 8:**
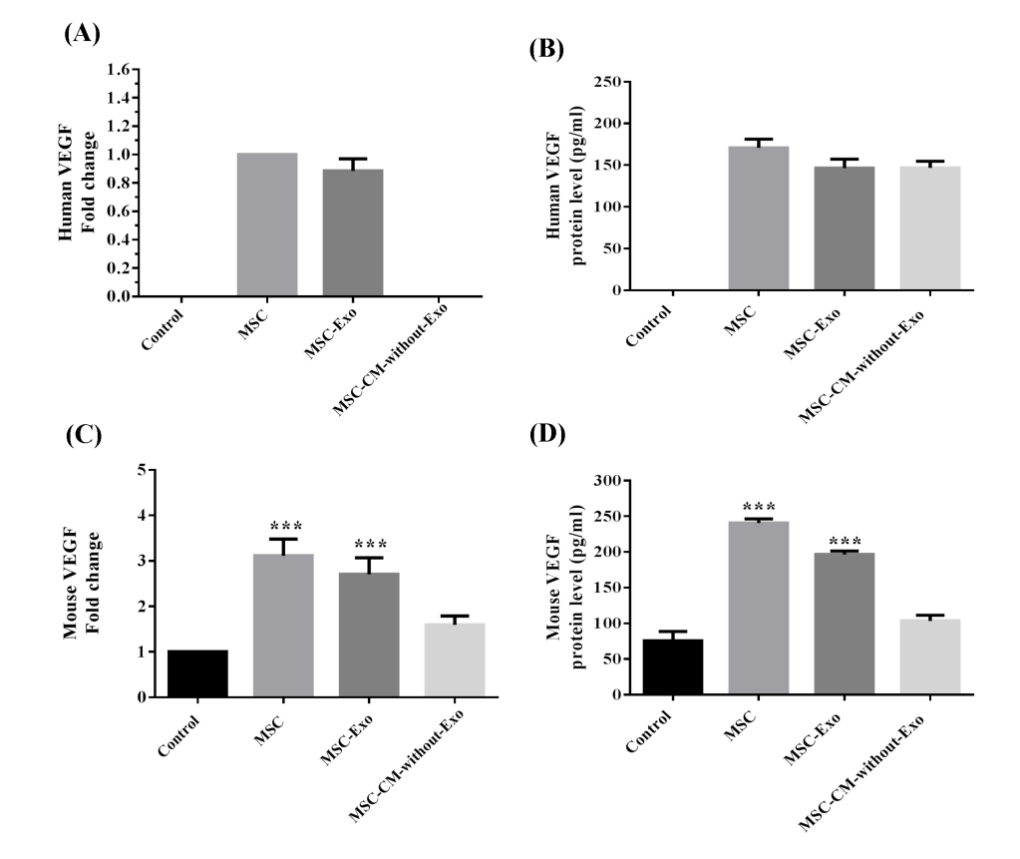
Human and mouse VEGF mRNA and protein expression in the lysed supernatant of mouse islet cells co-cultured with MSCs, MSC-Exo, and MSC-CM-without-Exo. ***p< 0.001. MSC: mesenchymal stem cell; MSC-Exo: MSC-derived exosomes; MSC-CM-without-Exo: MSC-conditioned medium without exosomes

VEGF expression was investigated given its importance in the survival and function of islets. Human- and mouse-specific VEGF protein and mRNA expression levels were measured in all groups following the three-day culture period. Human-specific VEGF protein or mRNA was not detected neither in the culture medium nor in the lysed islet cells in the control group, while the VEGF protein was present in the other three groups. The human VEGF protein levels in the islet supernatants were highest when co-cultured with MSCs, but also elevated when co-cultured with MSC-Exo and MSC-CM-without-Exo. Human VEGF mRNA was detectable only following co-culture with MSCs or MSC-Exo, but not with MSC-CM-without-Exo (Figure 8A, B(Fig. 8)), suggesting that MSC-Exo possibly entered the islets and released the cargo of various RNA and protein including VEGF mRNA into the islets.

The evaluation of mouse VEGF mRNA and protein revealed a significant increment in VEGF protein and mRNA levels in the islets co-cultured with MSC-Exo and MSC, but not in the MSC-CM-without-Exo group relative to the control. It could be proposed that exosomes, through the delivery of their contents, induce the expression and secretion of mouse VEGF in islet cells (Figure 8C, D(Fig. 8)).

### Mouse islet function during the cultureperiod

**Figure 9:**
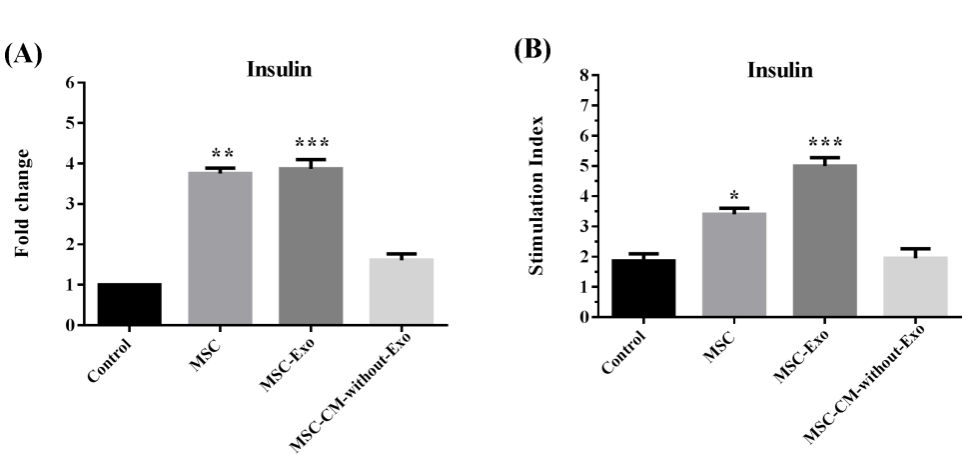
(A) Insulin mRNA and (B) glucose stimulation secretions in mouse islets co-cultured with MSCs, MSC-Exo, and MSC-CM-without-Exo. *p<0.05, **p<0.01, and ***p< 0.001. MSC: mesenchymal stem cell; MSC-Exo: MSC derived exosomes; MSC-CM-without-Exo: MSC-conditioned medium without exosomes

The function of the islets was evaluated in terms of insulin production and release. The transcription level of insulin was significantly higher in the islets cultured with MSC-Exo or MSCs compared with those cultured with MSC-CM-without-Exo. The protein secretion of insulin was also greater in the MSC-Exo and MSC groups, but not in the MSC-CM-without-Exo treatment. The MSC-Exo induced a more significant effect relative to the MSC group, suggesting that exosomes are the mediators of the paracrine function of MSCs in the promotion of islet function (Figure 9(Fig. 9)).

For additional results see Supplementary data.

## Discussion

Islet transplantation has aroused much interest among the therapeutic approaches for type 1 diabetes. However, loss of isolated islets and beta-cell dysfunction during the culture period are critical problems that affect successful transplantation outcomes (Bruni et al., 2014[4]; Pirjali et al., 2013[27]). Currently, isolated islets are incubated in culture media for 24-72 hours ahead of transplantation, during which the cell quality is evaluated, the recipient is prepared, immunosuppressive drugs are administered, and/or the isolated islets are transported to other centers. Previous studies have shown that isolated islets are faced by hypoxia and oxidative stress during the culture period (Keshtkar et al., 2020[18], 2019[19]; Zheng et al., 2012[45]) and the initial post-transplantation days, leading to islet death, particularly via the mitochondria apoptosis pathway (Miao et al., 2006[23]; Padmasekar et al., 2013[26]). 

Mesenchymal stem cells are protective and supportive cells that have been reported to be cultured or transplanted with islets (de Souza et al., 2017[8]). However, due to the possibility of tumorigenicity and other side effects of MSCs in clinical applications, novel therapeutic strategies must be sought (Nie et al., 2018[25]). Exosomes derived from MSCs appear to be a potentially safe and effective replacement for MSCs in regenerative medicine (Valadi et al., 2007[40]). The anti-apoptotic effect of MSC-Exo on other cells has been confirmed; MSC-Exo protected hepatocytes from acetaminophen-induced injury through the upregulation of the Bcl-xL anti-apoptotic gene as well as the Cyclin D1 proliferation gene (Tan et al., 2014[39]). Human tubular epithelial cells have also been protected from cisplatin-induced apoptosis by MSC-Exo via the augmentation of Bcl-xL and Bcl2 expression and the downregulation of caspase 1, caspase 8, and lymphotoxin alpha (Bruno et al., 2012[5]). Cui et al. reported that MSC-Exo significantly decreased the percentage of apoptotic cells and rescued cell viability in the myocardial H9c2 cell line under hypoxic condition following the downregulation of Bax and caspase-3 along with the upregulation of Bcl-2 and Cyclin D1 secondary to the activation of the Wnt/β-catenin pathway (Cui et al., 2017[7]). Recently, it was found that MSC-CM with exosomes protected neonatal porcine islet cell clusters (NICC) from hypoxia-induced cell death and improved their viability and function when compared with MSC-CM-without exosomes (Nie et al., 2018[25]). However, it is not known whether exosomes alone can replace MSCs for NICC preservation. Moreover, no study had previously investigated the effect of MSC-Exo on mature islets. In the present study, we demonstrated that MSC-Exo promoted islet viability and inhibited apoptosis as evidenced by live/dead staining and the TUNEL assay. These findings were confirmed by altered gene expression levels. We observed decreased expression of BAD and BAX, which are involved in the promotion of the intrinsic apoptosis pathway, and increased expression of BCL-2, which is regarded as an anti-apoptotic marker. The BAX/BCL-2 ratio, as a key regulator of the intrinsic apoptosis pathway, was also reduced in the presence of MSC-Exo (Kaviani et al., 2019[14][16][15]). Besides, the upregulation of PI3K was observed in islets co-cultured with exosomes, which may partially account for the effect of MSC-Exo on islet survival since PI3K can accelerate cell survival via the activation of Akt and the inhibition of BAD and BAX (Downward, 2004[9]). The PI3K signaling pathway is also known to be a critical regulator of the survival and inhibition of PI3K-induced islet death (Aikin et al., 2006[1]). A similar result was observed in islets co-cultured with MSCs, but not in the islets cultured in MSC-CM-without-Exo. In fact, exosomes were as effective as parent MSCs in the protection of mouse islets, while conditioned media without exosomes could not offer complete protection of islets against apoptosis. These findings suggest that the anti-apoptotic and pro-survival effects of MSC-Exo are probably mediated via the cross-talk between exosomes and islet cells. 

According to the literature, pancreatic human islets produce VEGF and its receptors (Sigrist et al., 2003[38]). Besides, exogenous VEGF treatment can protect islets against cell death both during the culture period and after transplantation (Cross et al., 2007[6]; Sigrist et al., 2003[38]). The addition of VEGF to encapsulated islets improved their viability and functionality in diabetic and healthy mice over 28 days following transplantation (Sigrist et al., 2003[38]). Also, the transfection of VEGF-expressing adenovirus in transplanted mouse islets ameliorated survival and controlled hypoglycemia on the first post-transplantation day (Cross et al., 2007[6]), confirming that VEGF has a pro-survival effect on islets during the culture period and early after transplantation. This effect of VEGF was found to be independent of its revascularization effect because revascularization initiated three or four days after transplantation (Cross et al., 2007[6]; Sigrist et al., 2003[38]). Moreover, MSC studies have elucidated that the paracrine function of these cells in the promotion of islet survival and cell membrane integrity is mediated by the secretion of various growth factors, especially VEGF. Yamada et al. reported that VEGF is influential in the MSC-related improvements in islet survival (Yamada et al., 2014[42]). In that study, the inhibition of VEGF by bevacizumab led to significantly decreased islet viability in co-cultured MSCs, suggesting that the anti-apoptotic effect of MSCs is attributed to VEGF (Yamada et al., 2014[42]). Jung et al. demonstrated that islets cultured in direct contact with MSCs had a significant increase in VEGF protein production, which contributed to islet survival and function (Jung et al., 2011[13]). In our study, the increment of mouse islets was associated with a significant decrement of apoptosis and increment of islet survival, suggesting that exosomes are the mediators of a critical paracrine function of MSCs, providing protection against apoptosis-induced islet cell death. 

The cytoprotective effect of exosomes is mediated by the delivery of various proteins, mRNA, and miRNA to target cells (Keshtkar et al., 2018[17]). It has been demonstrated that MSC-Exo contain unique proteins that are involved in cell-cell and cell-matrix attachments, extracellular matrix modulation, gap junction assembly, and the inhibition of the intrinsic apoptosis pathway. Also, MSC-Exo contain various transcription factors involved in the regulation of cell survival, differentiation, proliferation, and metabolic pathways. In our study, the human VEGF protein level in the MSC-Exo was almost equal to the level in MSC-CM-without-Exo, proposing that nearly half of the VEGFs were released and carried in the exosomes and the remaining half was released in soluble form. The presence of human VEGF mRNA and protein in the islets cultured with MSC-Exo was also remarkable. Based on these results, it is suggested that the anti-apoptotic and pro-survival effects of MSC-Exo are at least partially attributed to the transfer of human VEGF mRNA to the islet cells, which induced the production of mouse VEGF mRNA and protein, thereby preserving the islets. However, the precise mechanisms remain unclear. 

One of the key points for achieving a successful transplantation is to supply viable islets with proper, functional beta cells. The potential effect of MSCs on beta cell function in terms of insulin secretion is mediated through the release of a set of growth factors including VEGF. Moreover, it has been indicated that insulin is co-expressed with VEGF protein (Cross et al., 2007[6]), and the overexpression of VEGF in mouse islets also enhances the insulin content and improves normoglycemia in transplanted islets (Zhang et al., 2004[44]). In the present study, MSC-Exo augmented insulin gene expression and protein secretion in the mouse islets. The effect of MSC-Exo was slightly superior to that of MSCs, suggesting that human VEGF transfer via exosomes might contribute to the promotion of islet insulin secretion. On the other hand, it has been established that MSCs in direct contact with islets improve their function through adhesion molecules (Montanari et al., 2017[24]) and the production of extracellular matrix and annexin A-1 (Arzouni et al., 2017[2]). Annexin A1 is a ligand activated by MSCs that binds to G protein coupled receptors (GPCRs) of islets and enhances glucose-stimulated insulin secretion (Rackham et al., 2016[30]). The study of Riazifar et al. demonstrated that MSC-Exo contain a large amount of annexin A1 (Riazifar et al., 2019[31]). Therefore, it is possible that the delivery of various heterogeneous proteins and mRNA by MSC-Exo promoted the secretory function of islets even better than parent MSCs.

Taken together, our results demonstrated that MSC-Exo could not only improve the viability of isolated mouse islets but also enhanced their function. Moreover, the MSC-Exo were more effective than MSC-CM-without-Exo, suggesting that the VEGF, especially in its mRNA form, is one of the critical paracrine factors that fulfill a principal role in mitigating islet death and dysfunction. Interestingly, MSC-Exo were as effectual as parent MSCs in reducing apoptosis, increasing viability, and improving islet function. Albeit the protective effects of direct and indirect co-cultured MSCs and/or their conditioned media for improving islet quality have previously been reported, to the best of our knowledge, this is the first study illustrating that exosomes derived from MSCs are as effective as the parent MSCs in reducing islet death and promoting islet function. However, further *in vitro *and* in vivo* studies are necessary for obtaining a more comprehensive understanding of this phenomenon. 

These results suggest that MSC-Exo are the main paracrine therapeutic mediators of MSCs and exemplifies their potential for use in safe, effective, cell-free therapy. Indeed, in contrast to whole MSCs, MSC-Exo are easier to maintain and manage along; they are also safer owing to a lower amount of membrane-bound proteins and a lack of direct tumorigenesis ability (Keshtkar et al., 2018[17]). 

## Conclusion

In conclusion, MSC-Exo were as effective as the parental MSCs in improving islet survival and function during the culture period. Furthermore, the transfer of VEGF and its mRNA by exosomes might act as a crucial paracrine factor that mediates the mentioned cytoprotective effects.

## Acknowledgements

The authors are grateful to the Transplant Research Center of Shiraz University of Medical Sciences (Shiraz, Iran) for their financial support. 

## Funding

This work was sponsored by the Iran National Science Foundation (INSF; grant number 94808805) and was conducted at the Transplant Research Center of Shiraz University of Medical Sciences.

## Conflict of interest

The authors declare that they have no conflicts of interest.

## Authors’ contributions

S.K. and N.A. designed the study. S.K., M.K., F.S., and M.H.A. performed the experiments. M.H.G. and N.A. coordinated the study; S.K., M.K., and F.S. analyzed the data; S.K. drafted the manuscript; I.H.A.A., M.H.G., and N.A. interpreted the data and revised the manuscript; N.A. prepared the latest revision of the manuscript. All authors read and approved the final manuscript.

## Supplementary Material

Supplementary data
