# Exosomes derived from human mesenchymal stem cells preserve mouse islet survival and insulin secretion function

**DOI:** 10.17179/excli2026-9390

**Published:** 2026-03-05

**Authors:** Somayeh Keshtkar, Maryam Kaviani, Fatemeh Sabet Sarvestani, Mohammad Hossein Ghahremani, Mahdokht Hossein Aghdaei, Ismail H. Al-Abdullah, Negar Azarpira

**Affiliations:** 1 Department of Molecular Medicine, School of Advanced Technologies in Medicine, Tehran University of Medical Sciences, Tehran, Iran; 2 Transplant Research Center, Shiraz University of Medical Sciences, Shiraz, Iran; 3 Department of Translational Research and Cellular Therapeutics, Diabetes and Metabolism Research Institute, Beckman Research Institute of City of Hope, Duarte, CA/USA

## ⁯Corrigendum

Corrigendum to original article: https://doi.org/10.17179/excli2020-2451

[EXCLI Journal 2020;19:1064-1080]; PMID: 33013264; PMCID: PMC7527509

After publication of the above-cited article, it was brought to the attention of the Editors that Figure 1A contained repeated structures in the upper right corner, which could give the impression of digital alteration.

Upon inquiry, the authors promptly provided the original, unprocessed version of Figure 1A, which includes a scale bar located in the upper right corner. The image is intended solely to illustrate mouse pancreatic islets after digestion. The prior removal of the scale bar does not affect the scientific content, interpretation, or conclusions of the article. In order to ensure full transparency and to avoid any potential ambiguity, the unprocessed version of Figure 1A is reproduced below.

(Fig. 1)

*The Editors*

*EXCLI Journal*

## Figures and Tables

**Figure 1:**
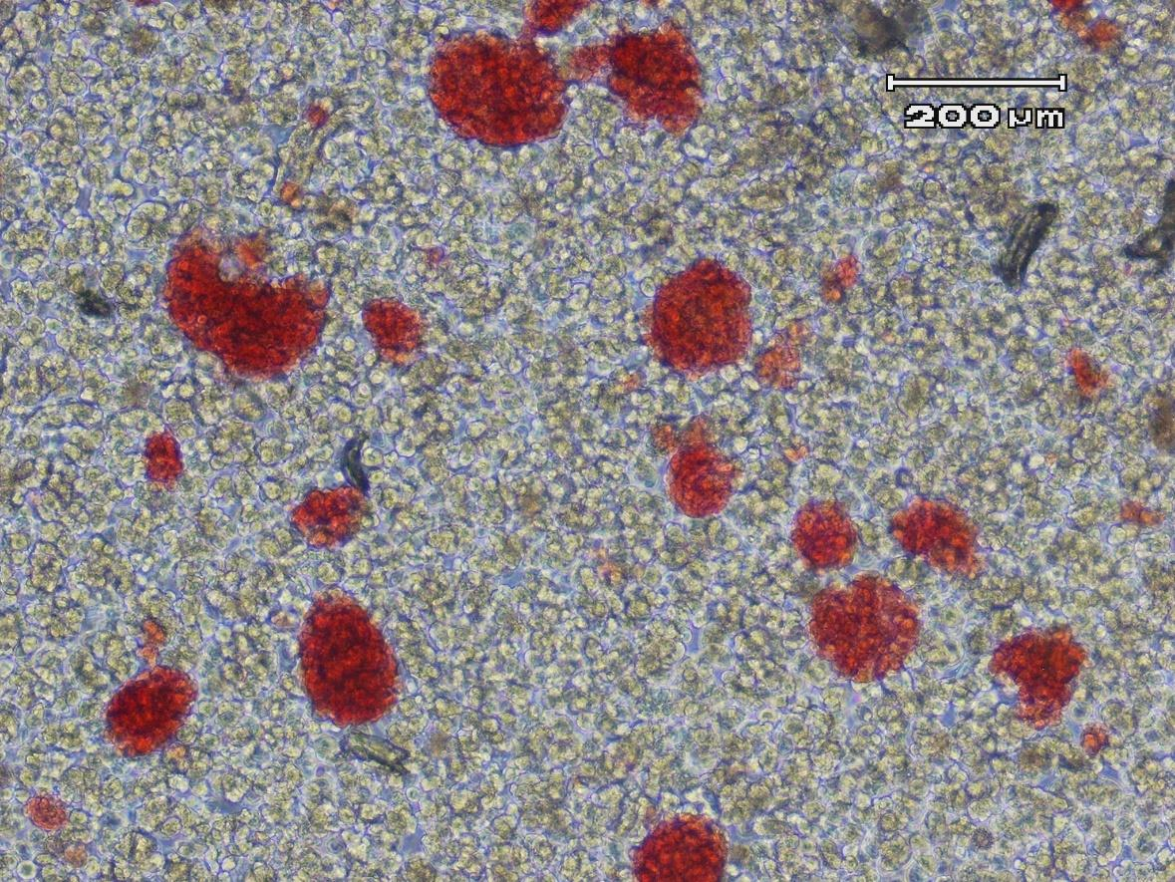
Unprocessed Figure 1A from the above-cited article

