# Supplementary material for: Exosomes derived from human mesenchymal stem cells preserve mouse islet survival and insulin secretion function
Source: EXCLI J. 2020 Aug 3;19:1064–80. doi: 10.17179/excli2020-2451 (PMC7527509; doi:10.17179/excli2020-2451)
Supplement: Supplementary data [file EXCLI-19-1064-s-001.pdf]

EXCLI Journal 2020;19:1064-1080 – ISSN 1611-2156
Received: May 23, 2020, accepted: July 20, 2020, published: August 03, 2020

Supplementary data to:

EXOSOMES DERIVED FROM HUMAN MESENCHYMAL STEM CELLS PRESERVE MOUSE ISLET SURVIVAL AND INSULIN SECRETION FUNCTION

Somayeh Keshtkar[1,2], Maryam Kaviani[2], Fatemeh Sabet Sarvestani[2], Mohammad Hossein Ghahremani[1], Mahdokht Hossein Aghdaei[2], Ismail H. Al-Abdullah[3], Negar Azarpira[2*]

[1] Department of Molecular Medicine, School of Advanced Technologies in Medicine, Tehran University of Medical Sciences, Tehran, Iran
[2] Transplant Research Center, Shiraz University of Medical Sciences, Shiraz, Iran
[3] Department of Translational Research and Cellular Therapeutics, Diabetes and Metabolism Research Institute, Beckman Research Institute of City of Hope, Duarte, CA/USA

* **Corresponding author:** Negar Azarpira, Transplant Research Center, Shiraz University of Medical Sciences, Shiraz, Iran, Telephone number: +98-71-36281529, Fax: +98-711-6473954; E-mail: negarazarpira@gmail.com

http://dx.doi.org/10.17179/excli2020-2451

This is an Open Access article distributed under the terms of the Creative Commons Attribution License (http://creativecommons.org/licenses/by/4.0/).

EXCLI Journal 2020;19:1064-1080 – ISSN 1611-2156
Received: May 23, 2020, accepted: July 20, 2020, published: August 03, 2020

Supplementary Table 1: Raw data of islet viability staining by fluorescein diacetate (green) for living cells and propidium iodide (red) for dead cells. The viability rate was expressed by the percentage of green area to the total area within intact islet. The raw data are related to **Figure 4** in the main text.

**Percentage of green area to the total area within each islet**			
**Control**	**MSC**	**MSC-Exo**	**MSC-CM-without-Exo**
75	100	100	100
50	100	100	100
100	100	100	50
0	100	100	25
0	100	100	100
0	100	100	100
100	100	100	100
100	100	100	100
100	100	100	75
0	100	100	50
75	100	100	0
100	100	100	0
	100	100	100
	100		100
	100		100
	100		100
	100		
	100		

MSC: mesenchymal stem cell; MSC-Exo: MSC-derived exosomes; MSC-CM-without-Exo: MSC-conditioned medium without exosomes

EXCLI Journal 2020;19:1064-1080 – ISSN 1611-2156
Received: May 23, 2020, accepted: July 20, 2020, published: August 03, 2020

Supplementary Table 2: Raw data of TUNEL-positive (apoptotic) cells. Apoptotic cells were green florescent and the nuclei were stained blue by DAPI dye. The percentage of apoptotic islets was expressed by the percentage of TUNEL-positive cells out of all nuclei within each islet. The raw data are related to Figure 5 in the main text.

Percentage of TUNEL-positive cells (green) to total nuclei (blue)											
**Control**			**MSC**			**MSC-Exo**			**MSC-CM-without-Exo**		
**DAPI (N)**	**Green (N)**	**Green/DAPI *100**	**DAPI (N)**	**Green (N)**	**Green/DAPI *100**	**DAPI (N)**	**Green (N)**	**Green/DAPI *100**	**DAPI (N)**	**Green (N)**	**Green/DAPI *100**
67	15	22.388	38	1	2.631	60	0	0	110	34	30.909
24	0	0	43	2	4.651	15	0	0	105	14	17.142
29	0	0	32	0	0	17	0	0	93	18	19.354
78	35	44.871	22	0	0	96	0	0	55	14	25.454
38	21	55.263	10	0	0	35	1	2.857	95	20	21.052
85	54	63.529	25	1	4	85	3	3.529	135	20	14.814
55	39	70.909	26	0	0	78	4	5.128	30	4	13.333
168	65	38.690	30	0	0	42	3	7.142	40	25	62.5
25	12	48	160	2	1.25	106	3	2.830	47	2	4.255
37	7	18.918	42	3	7.142	42	2	4.761	50	18	36
43	1	2.325	87	8	9.195	177	6	3.389	40	25	62.5
100	68	68	194	3	1.546	82	6	7.317	24	4	16.666
			140	14	10				24	2	4.167

MSC: mesenchymal stem cell; MSC-Exo: MSC-derived exosomes; MSC-CM-without-Exo: MSC-conditioned medium without exosomes; N: Number

EXCLI Journal 2020;19:1064-1080 – ISSN 1611-2156
Received: May 23, 2020, accepted: July 20, 2020, published: August 03, 2020

Supplementary Table 3: Raw data of gene expression of BCL-2, BAX, BAD, PI3K, VEGF and insulin in mouse islets. GAPDH was used as the housekeeping gene. The raw data are related to **Figure 6 (A-D), Figure 8C, and Figure 9A** in the main text.

Gene (CT)/Groups	Control			MSC			MSC-Exo			MSC-CM-without-Exo		
GAPDH	16.34	16.07	16.8	16.6	17.44	15.77	16.26	16.45	16.04	17.92	16.49	16.1
	16.34	16.13	16.81	16.94	17.96	16.97	16.27	16.47	16.14	18.23	16.48	18
BAX	20.08	19.17	21.57	22.98	23.51	21.56	21.85	21.77	21.1	22.16	20.42	21.56
	19.89	20.41	21.6	22.02	22.57	21.05	20.78	21.55	22.45	22.35	20.55	21.33
BAD	24.82	23.44	23.67	25.88	26.42	26.02	25.42	25.44	24.85	26.64	24.75	25.34
	24.38	23.48	23.6	25.74	26.24	25.95	24.89	25.33	25.39	27.38	25.48	25.39
BCL-2	29.27	27.97	28.75	26.9	27.48	26.63	26.15	27.33	25.73	28.81	28.42	28.36
	28.49	28.07	28.18	27.17	27.98	26.73	26.22	26.88	26.57	29.97	28.16	27.83
PI3K	27.26	28.52	27.78	26.4	27.95	26.57	26.2	26.55	26.45	28.42	26.78	27.3
	28.88	27.37	28.35	27	27.25	26.35	26.46	26.06	25.98	27.95	26.36	27.22
VEGF	19.13	19.14	20.03	18.24	18.36	17.94	17.9	18.22	16.92	20.45	18.78	19.24
	18.64	19.1	20.02	18.11	19.45	17.57	17.5	18.34	18.05	20.85	19.06	18.66
Insulin	12.09	14.08	15.46	13.31	13.07	11.98	11.49	12.11	11.3	14.78	13.29	14
	11.49	13.65	15.97	13.62	13.05	11.97	11.61	11.93	11.8	15.11	12.62	13.75

BAD: BCL-2-associated agonist of cell death; BAX: BCL-2 associated X: BCL-2: B-cell lymphoma-2; GAPDH: Glyceraldehyde 3-phosphate dehydrogenase; PI3K: Phosphoinositide 3-kinase MSC: mesenchymal stem cell; MSC-Exo: MSC-derived exosomes; MSC-CM-without-Exo: MSC-conditioned medium without exosomes

EXCLI Journal 2020;19:1064-1080 – ISSN 1611-2156
Received: May 23, 2020, accepted: July 20, 2020, published: August 03, 2020

Supplementary Table 4: Raw data of the VEGF concentration in MSC-Exo compared with MSC-CM-without-Exo after ultracentrifugation. The raw data are related to **Figure 7** in the main text.

Human VEGF protein level (pg/ml)	
**MSC-Exo**	**MSC-CM-without-Exo**
507.535	507.535
562.363	662.363
497.450	697.450
558.245	558.245
593.550	593.550
516.580	616.580

VEGF: Vascular endothelial growth factor; MSC-Exo: Mesenchymal stem cell derived exosomes; MSC-CM-without-Exo: MSC-conditioned medium without exosomes

Supplementary Table 5: Raw data of gene expression of human VEGF stimulation in mouse islets co-cultured with MSC, MSC-Exo, and MSC-CM-without-Exo. The raw data are related to **Figure 8A** in the main text.

Gene (CT)/Groups	Control			MSC			MSC-Exo			MSC-CM-without-Exo		
**GAPDH**	ND	ND	ND	19.53	19.78	19.44	18.96	19.77	18.09	ND	ND	ND
	ND	ND	ND	18.94	18.85	19.7	18.93	18.16	18.29	ND	ND	ND
**VEGF**	ND	ND	ND	22.98	21.255	21.08	21.48	19.33	18.4	ND	ND	ND
	ND	ND	ND	21.27	21.194	21.96	18.96	19.09	18.25	ND	ND	ND

ND: Not detected; VEGF: Vascular endothelial growth factor; MSC: Mesenchymal stem cell; MSC-Exo: MSC-derived exosomes; MSC-CM-without-Exo: MSC-conditioned medium without exosomes

Supplementary Table 6: Raw data of mouse and human VEGF protein in the supernatant of mouse islets co-cultured with MSC, MSC-Exo, and MSC-CM-without-Exo. The raw data are related to **Figure 8B and 8D** in the main text.

Human VEGF protein level (pg/ml)				Mouse VEGF protein level (pg/ml)			
**Control**	**MSC**	**MSC-Exo**	**MSC-CM-without-Exo**	**Control**	**MSC**	**MSC-Exo**	**MSC-CM-without-Exo**
**0.**	170.840	158.000	137.000	50.840	250.040	188.000	98.000
**0.**	181.010	137.400	149.030	81.010	231.580	197.400	119.030
**0.**	160.100	144.580	153.220	95.100	240.100	204.580	93.220
**0.**	162.954	145.063	159.240	41.495	219.960	200.408	101.910
**0.**	184.615	134.945	143.412	102.254	233.130	208.057	78.050
**0.**	164.850	160.032	138.750	83.201	269.180	179.325	130.576

VEGF: Vascular endothelial growth factor; MSC: Mesenchymal stem cell; MSC-Exo: MSC-derived exosomes; MSC-CM-without-Exo: MSC-conditioned medium without exosomes

Supplementary Table 7: Raw data of insulin concentration (ng/ml) after 2.8 mM and 20 mM glucose stimulation in mouse islets co-cultured with MSC, MSC-Exo, and MSC-CM-without-Exo. Glucose stimulation index (GSI) was calculated by dividing the value of insulin secretion in 20 mM glucose medium into the value obtained in the 2.8 mM glucose medium. The raw data are related to **Figure 9B** in the main text.

Control		MSC		MSC-Exo		MSC-CM-without-Exo		GSI (20 mM/2.8 mM)			
**2.8 mM**	**20 mM**	**2.8 mM**	**20 mM**	**2.8 mM**	**20 mM**	**2.8 mM**	**20 mM**	**Control**	**MSC**	**MSC-Exo**	**MSC-CM-with-out-Exo**
1.07	1.416	2.48	8.07	4.04	16.45	2.15	5.01	1.323	3.254	4.071	2.330
2.219	4.73	3.059	10.56	2.24	10.48	2.94	7.3	2.131	3.452	4.678	2.482
2.13	4.59	2.62	7.93	2.8	14.7	2.99	3.07	2.155	3.026	5.25	1.029
1.02	1.31	3.02	12.558	2.68	14.59	2.47	6.28	1.284	4.158	5.444	2.543
1.59	3.849	3.18	9.96	3.37	18.72	2.97	4.09	2.420	3.132	5.554	1.377

MSC: mesenchymal stem cell; MSC-Exo: MSC-derived exosomes; MSC-CM-without-Exo: MSC-conditioned medium without exosomes; GSI: Glucose stimulation index
